# Benefits of the Global Integration Method (Método de Integração Global - MIG) in functional priorities of parents of Brazilian children and adolescents with autism spectrum disorder

**DOI:** 10.1186/s12887-025-05422-0

**Published:** 2025-01-30

**Authors:** Amanda Aparecida Alves Cunha Nascimento, Deisiane Oliveira Souto, Thalita Karla Flores Cruz, Arthur Felipe Barroso de Lima, Gabriela Silva Oliveira, Vitor Geraldi Haase

**Affiliations:** 1 Institute of Neurodevelopment, Cognition, and Inclusive Education (INCEI), Ribeirão das Neves, Belo Horizonte, MG Brazil; 2 Postgraduate Program in Neuroscience, Federal University of Minas Gerais, Belo Horizonte, MG Brazil; 3 Postgraduate Program in Rehabilitation Sciences, Federal University of Minas Gerais, Belo Horizonte, MG Brazil; 4 Department of Physical Education, Physiotherapy, and Occupational Therapy, Federal University of Minas Gerais, Belo Horizonte, MG Brazil; 5 Postgraduate Program in Psychology, Cognition and Behavior, Federal University of Minas Gerais, Belo Horizonte, Brazil; 6 Graduate Program in Rehabilitation Sciences, Physical Therapy Department, School of Physical Education, Physical Therapy and Occupational Therapy, Universidade Federal de Minas Gerais, Av. Pres. Antônio Carlos, 6627 Campus – Pampulha, Minas Gerais Belo Horizonte, 31270-901 Brazil

**Keywords:** Functional priorities, Children, Adolescents, Autism spectrum disorder

## Abstract

**Background:**

Understanding the priorities of parents of children and adolescents with autism spectrum disorder (ASD) is crucial for implementing evidence-based programs. This study aims to identify the functional priorities of parents of Brazilian children and adolescents with ASD, analyze variations in priorities according to the levels of support and age groups of the participants, and categorize the goals according to the categories of the International Classification of Functioning, Disability, and Health (ICF). Additionally, this study aimed to evaluate changes in parents’ performance and satisfaction with functional priorities after intervention with the Global Integration Method (Métodode Integração Global - MIG).

**Methods:**

A total of 241 children/adolescents with ASD (mean age, 6.92 ± 3.61 years) were recruited from different regions of Brazil. 76% (76%) were male, and 40.7% were classified as having support level 2. The Canadian Occupational Performance Measure was administered to parents/caregivers to identify their priorities for their children and to assess changes in performance and satisfaction with priorities after intervention with MIG. The MIG protocol consisted of functional task training in a naturalistic environment (City of Tomorrow) combined with the use of a flexible therapeutic suit (MIG Flex) and was conducted for 3 months, five times a week, for 3–4 h per day. Descriptive statistics were used to provide the priority profile. Pre- and post-intervention data were analyzed using paired t-test.

**Results:**

Parents established 1,203 functional priorities. Activities of daily living, behavioral difficulties, communication, play, and social interactions were the main functional priorities in the perception of parents/caregivers. The profiles of functional priorities were similar between the different levels of support and age groups. Approximately 64% of the priorities were classified in the activity domain of the ICF. In general, the MIG program resulted in significant improvements in performance and satisfaction for the majority of functional priorities (*p* < 0.05).

**Conclusion:**

Activities of daily living appear to be the main priority of parents of children and adolescents with ASD, regardless of the level of support or age group. The MIG program has been associated with improvements in performance and satisfaction across several of the functional priorities identified by parents.

## Introduction

Autism spectrum disorder (ASD) comprises a heterogeneous group of neurodevelopmental conditions characterized by persistent deficits in the communication triad, social interactions, and restricted and stereotyped behaviors/interests, leading to significant functional impairment [1]. Generally, individuals with ASD may have co-occurring conditions, including motor disorders, sleep disturbances, epilepsy, genetic syndromes, and psychiatric disorders. [2, 3]. ASD is a complex and multifactorial condition with considerable phenotypic variability that potentially affects all domains of functionality as proposed by the International Classification of Functioning, Disability, and Health (ICF) [4, 5]. The ICF provides a standardized language for describing health and its related aspects, dividing into three main components: activity, participation, and body structures and functions, which interact with each other and with contextual factors to determine an individual’s functionality or disability. The interaction between these components is complex for most children with ASD; for instance, changes in body functions, such as communication difficulties, can influence the daily activities and social participation of someone with autism [6]. This comprehensive perspective allows for a deeper understanding of individual needs, promoting more effective and personalized interventions.

Given the variety of manifestations, strengths, weaknesses, most children are likely to require ongoing rehabilitation programs [7]. Parents and professionals have made efforts to identify effective evidence-based interventions for individuals with ASD [8]. Current scientific knowledge supports the implementation of therapies that are family-centered and incorporate collaboration between families and professionals [9, 10]. According to these approaches, the child and family should be involved in all aspects of decision-making, informing healthcare professionals of their priorities [11]. As children/adolescents and their families have needs in many areas, setting goals through collaboration between families and professionals is necessarily complemented by team-based intervention rather than by multiple goals from each discipline pursued independently. Thus, efforts are also needed to provide children/adolescents with ASD and their families with interdisciplinary care, as their complex needs cannot be adequately met without interaction among healthcare professionals [8].

Currently, few comprehensive interventions that emphasize family-centered practice and interdisciplinary coordination are available for children/adolescents with ASD and their families. One example is the Global Integration Method - MIG [8]. The Global Integration Method (Método de Integração Global - MIG) is an intervention program that utilizes the principles of family-professional collaboration and family-centered practice. MIG involves shared therapeutic decision-making with the family and encompasses intervention strategies capable of addressing impairments in all domains of the ICF. This integrated, intensive, and interdisciplinary approach was developed for children with ASD based on the best current evidence in pediatric rehabilitation [8].

Unlike traditional rehabilitation approaches, care in MIG is provided intensively and interdisciplinarily by a single team working collaboratively in one location. In MIG, the intensive approach is implemented in a naturalistic environment called the “City of Tomorrow” using a flexible therapeutic suit, the “MIG Flex” [8]. The “City of Tomorrow” consists of naturalistic learning environments, or units, that simulate real-life environments such as home, school, market, streets, and sports courts. Through the units of the “City of Tomorrow,” children have the opportunity to participate in inherently reinforcing activities while developing cognitive schemes and learning to use them in a concrete, contextualized, and relevant way to life. It is believed that interventions in children’s living environments or similar settings promote social learning and generalization [8]. 

**Fig. 1:**
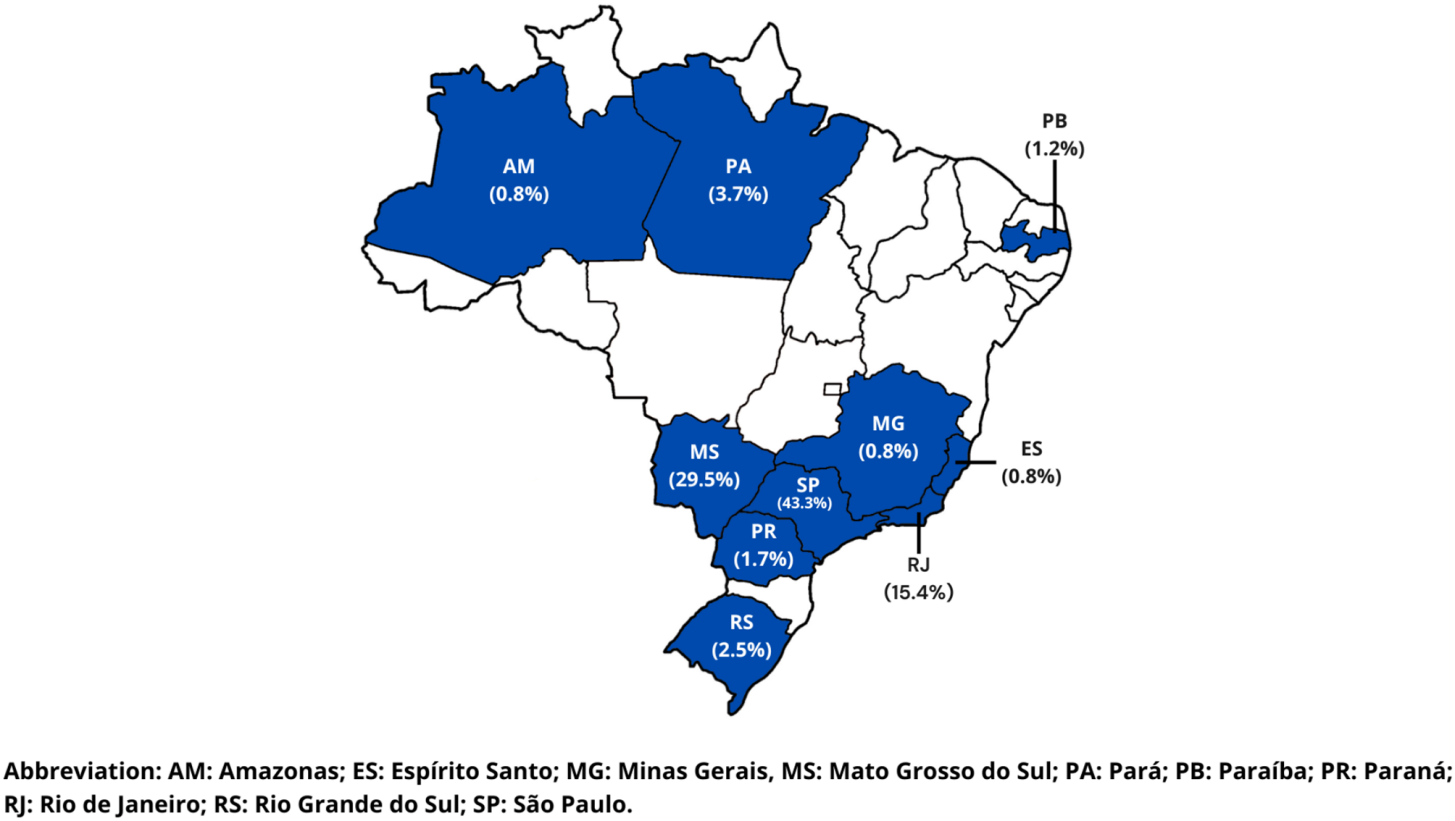
MIG program. **a**) therapeutic suit MIG Flex and **b**) different units of the City of Tomorrow

The activities within the “City of Tomorrow” are carried out using the MIG Flex. The MIG Flex is based on myofascial meridians. It primarily addresses motor alterations and muscular hypotonia often observed in individuals with ASD [8]. Prolonged use of the MIG Flex may aid in the transmission of adequate proprioceptive information to the central nervous system, which can lead to more precise execution of movements by patients [8]. This is an innovative aspect of the MIG program, suggesting potential for improving cognitive symptoms by reducing motor overload and freeing up processing resources for social cognitive learning. However, this benefit is currently hypothetical and requires further evaluation to establish its effectiveness. Figure 1 provides images of the (a) therapeutic suit MIG Flex and (b) different units of the City of Tomorrow. In MIG, the Canadian Occupational Performance Measure (COPM) is used to set rehabilitation goals, which reflect parents’ priorities.

Despite the importance of understanding and incorporating parental priorities into rehabilitation services, few therapeutic modalities have shown how their programs are derived from parental priorities for individuals with ASD [12]. Therapists must understand the priorities and needs of the child from the parents’ perspective, as they are the primary experts on the child’s abilities and needs [13]. Identifying parental priorities and incorporating them into intervention programs will increase parental acceptance, participation, and satisfaction, leading to better outcomes for children/adolescents [14]. Furthermore, knowing the functional priorities of children/adolescents and their families enables professionals to more effectively direct the assessment process to areas of highest priority, thus facilitating the planning of appropriate and efficient treatments.

Several studies have investigated the functional priorities of children and adolescents with ASD, and their families [7, 12, 14, 15]. The development of social communication was the highest intervention priority for the parents of 207 Iranian children with ASD. In a study by Pituch et al. [7], 90 parents of children with ASD from different countries (New Zealand, the USA, Australia, and Canada) identified priorities such as social skills, communication, academic skills, community living skills, vocational skills, and recreation/leisure skills. Communication, maladaptive behavior, and social interaction were the priorities found in the study by Rodger et al. [12], which involved 22 parents of Australian children with ASD. In a study by Spann et al. [15], social skills, communication, and activities of daily living (e.g., dressing and cooking) were identified as priorities for 45 parents of Spanish children with ASD.

To date, the studies conducted so far have not provided information on how parental priorities relate to the level of support and age of children/adolescents with ASD. Additionally, the majority of studies have involved children/adolescents from developed countries. There is a scarcity of this type of research in developing countries, such as Brazil. Therefore, the present study aims to: (1) Identify the functional priorities of families of Brazilian children and adolescents with ASD, (2) Determine differences in functional priorities according to the level of support and age range of the participants, (3) Categorize the functional priorities according to the structure of the ICF, (4) Evaluate changes in parental performance and satisfaction with functional priorities after intervention with the MIG program.

## Methods

For this study, a temporal cut was used from an ongoing longitudinal research aimed at monitoring children and adolescents with ASD participating in the intervention program with the MIG. All research procedures were approved by the Human Research Ethics Committee of the Medical Sciences Faculty of Minas Gerais (Protocol No 72360923.9.0000.5134). Written informed consent was obtained from the parents/guardians of all children and adolescents with ASD before assessment. Children and adolescents who agreed to participate in the study provided their consent.

### Participants

Participants were recruited from 78 clinics located in different regions of Brazil that provide rehabilitation services to children and adolescents with neurodevelopmental disorders and offer the MIG program as an intervention modality. Currently, 410 children and adolescents with ASD are served by the MIG program and were invited to participate in the study. A total of 241 children/adolescents aged 1.8 − 18.2 years agreed to participate in this study; 76% were male, and 40.7% were classified as having support level 2. The characteristics of the participants are presented in Table 1. All participants were diagnosed with ASD according to the Diagnostic and Statistical Manual of Mental Disorders, 5th edition [16]. Participants did not have cognitive, behavioral, or clinical limitations that prevented them from following instructions and participating safely in the proposed MIG activities, according to parents’ report.

**Table 1:** Characteristics of the study participants

	M (SD)	
Age (years)	6.95 (3.69)	
Workload (weekly)	17.7 (3.19)	
n (%)		
Sex		
Female	183 (75.9)	
Male	58 (24.1)	
Support level		
Level 1	56 (25.9)	
Level 2	88 (40.7)	
Level 3	72 (33.3)	
Who responded to COPM		
Mother	199 (83.6)	
Father	30 (12.6)	
Grandparents	3 (1.3)	
Other family members	5 (2.1)	
Person with autism	1 (0.4)	
Maternal education		
Graduated	105 (44.3)	
Complete secondary/incomplete higher education	104 (43.9)	
Elementary school II complete/incomplete high school	15 (6.3)	
Elementary school I complete/incomplete elementary school II	7 (3)	
Illiterate/incomplete elementary school I	6 (2.5)	
Mother’s occupation		
Full-time (8 h per workday)	94 (39.5)	
Housewife	83 (34.9)	
Partial (4–6 h per workday)	61 (25.6)	

Most of the participants were recruited from clinics in the Southeast (61%) and Midwest (29%) regions. A small number of participants were recruited from the remaining regions (North = 4.6%, South = 4.1%, Northeast = 1.2%). Figure 1 shows the distribution of the participants by state. A predominance of the participants were from São Paulo (43.3%) and Mato Grosso do Sul (29.5%). In general, services for these children are financed by private health insurance according to Brazilian legislation [17]. Approximately 83.6% of the interviews with COPM were answered by mothers.

### Measurement instruments

Information about the functional properties of families was evaluated using the COPM [18]. COPM is a standardized instrument designed to identify and quantify functional priorities related to self-care (e.g., personal care, functional mobility, community management), productivity (e.g., paid or unpaid work, household management, school), and leisure (e.g., quiet recreation, active recreation, socialization) [19]. It consists of a semi-structured interview in which parents list their treatment priorities and assess levels of performance and satisfaction with their children’s performance on a scale from 1 (unable to perform the activity) to 10 (performs extremely well) [20]. In addition to being a reliable and valid instrument [20], the COPM has been used effectively by children with disabilities and their families [21]. The COPM can detect changes in performance over time and after an intervention [18–23].

### Intervention program MIG

The participants underwent three months of intervention with the MIG program, administered 3 to 5 times a week, with sessions lasting 3 to 4 h per day. In the MIG program, the process begins with the collaborative application of the COPM with the family to identify about three to five therapeutic goals. Subsequently, an interdisciplinary team, together with the family, develops a personalized therapeutic plan focused on the specific goals of each child/adolescent. The planning covers the different domains of the ICF that impact the achievement of the proposed goals. The targeted training to achieve these goals is conducted with the help of the MIG Flex therapeutic suit in a naturalistic environment called the “City of Tomorrow.” All necessary motor, cognitive, social, and psychological skills required to achieve the established goals are trained. The selection of professionals involved in the program is made according to the needs and demands of each child, and may include physiotherapists, occupational therapists, speech therapists, and psychologists. All sessions of the MIG program are conducted by professionals experienced in pediatric rehabilitation and trained in the MIG program.

### Procedures

Data were collected between August 2023 to May 2024. The COPM was administered collaboratively between the family and the evaluator, who was a professional with a background in occupational therapy and experience in administering this instrument. However, all professionals involved in the administration of COPM have undergone remote training to standardize its application. The evaluation of each participant was carried out at the rehabilitation clinic where they underwent the intervention program.

### Statistical analysis

The functional priorities identified by parents in the COPM application were categorized and grouped according to the categories of the American Occupational Therapy Association (AOTA) [24]. For example, the goal “I would like my child to hold a spoon with food and be able to bring it to his mouth” was coded as “Feeding.” The categorization of functional priorities according to AOTA categories was carried out by two independent researchers (DOS and GSO). Any disagreements were resolved by a third researcher (TKFC). The same procedure was adopted to group functional priorities according to the ICF domains. The Statistical Package for the Social Sciences (version 22.0) was used for data analysis. Descriptive statistics were used to describe the demographic characteristics of participants and summarize parental priorities for their children by category and subcategory. Chi-square tests were used to test the association between the functional priorities established by parents in COPM and the classification of support levels, as well as the age range of the participants. Participants were grouped into the following age ranges: 0–6 years (infancy and preschool age); 7 − 12 years (school age); and 13 − 18 years (adolescence). To assess the effects of the MIG program, the percentage of targets that achieved changes in the COPM score was initially calculated. The student’s t-test for paired samples was used to verify changes in performance and satisfaction of the participants after intervention with the MIG program. To do this, the overall average of all goals in the pre- and post-intervention assessments was calculated. Cohen’s d was used to assess the magnitude of the intervention program effects (large effect = 0.80, medium effect = 0.50, small effect = 0.20). Analyses with the t-test were performed for the 10 most frequent functional priorities, which are: (1) Feeding; (2) Personal Hygiene; (3) To wear; (4) Mental functions related to Behavior; (5) Use the toilet and perform personal hygiene; (6) Communication Management; (7) Exploratory playing; (8) Bathing and taking a shower; (9) Social Interaction (10) Formal education participation.

## Results

### Functional priorities

**Fig. 2:**
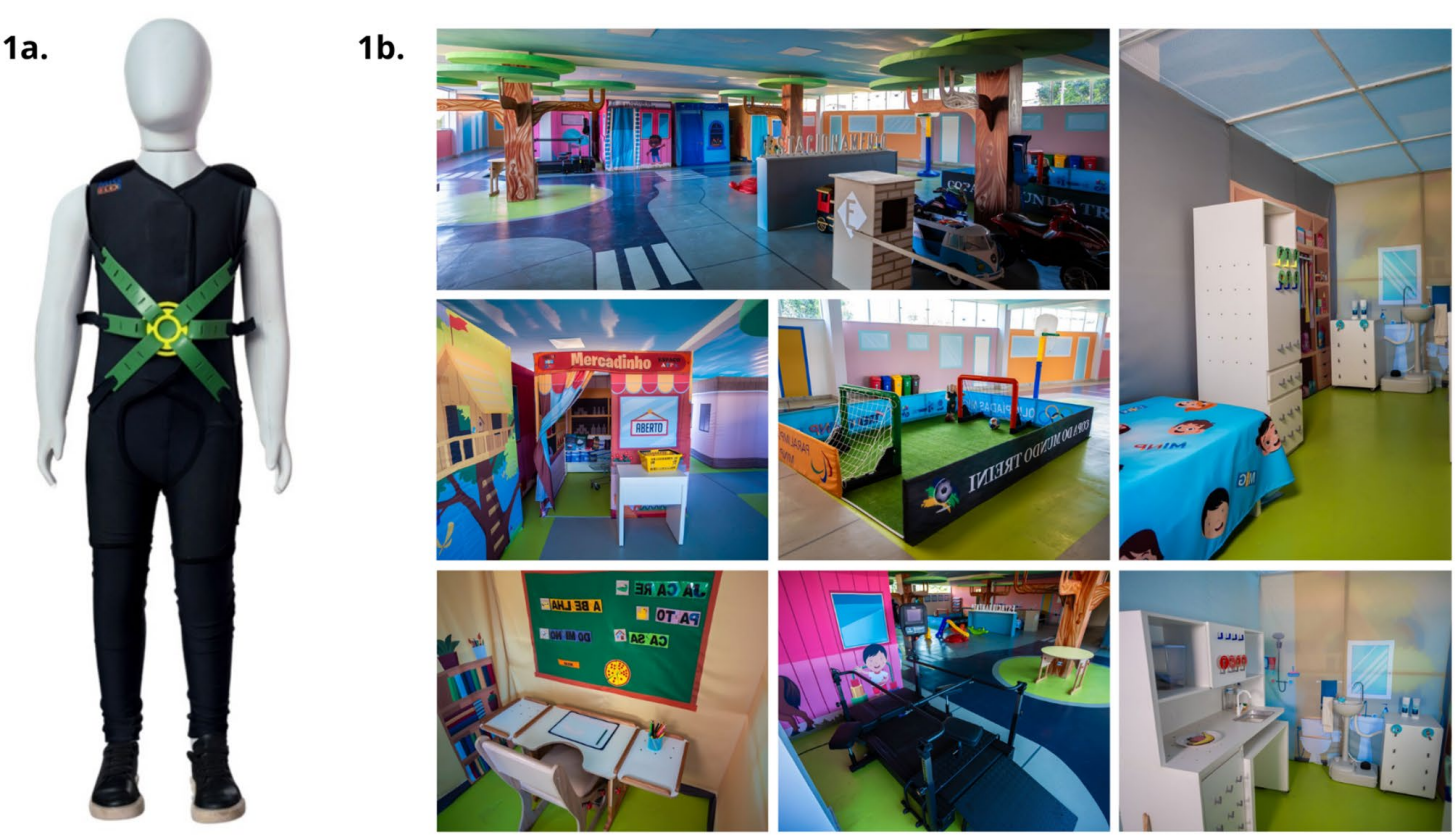
Distribution of participants by state

**Fig. 3:**
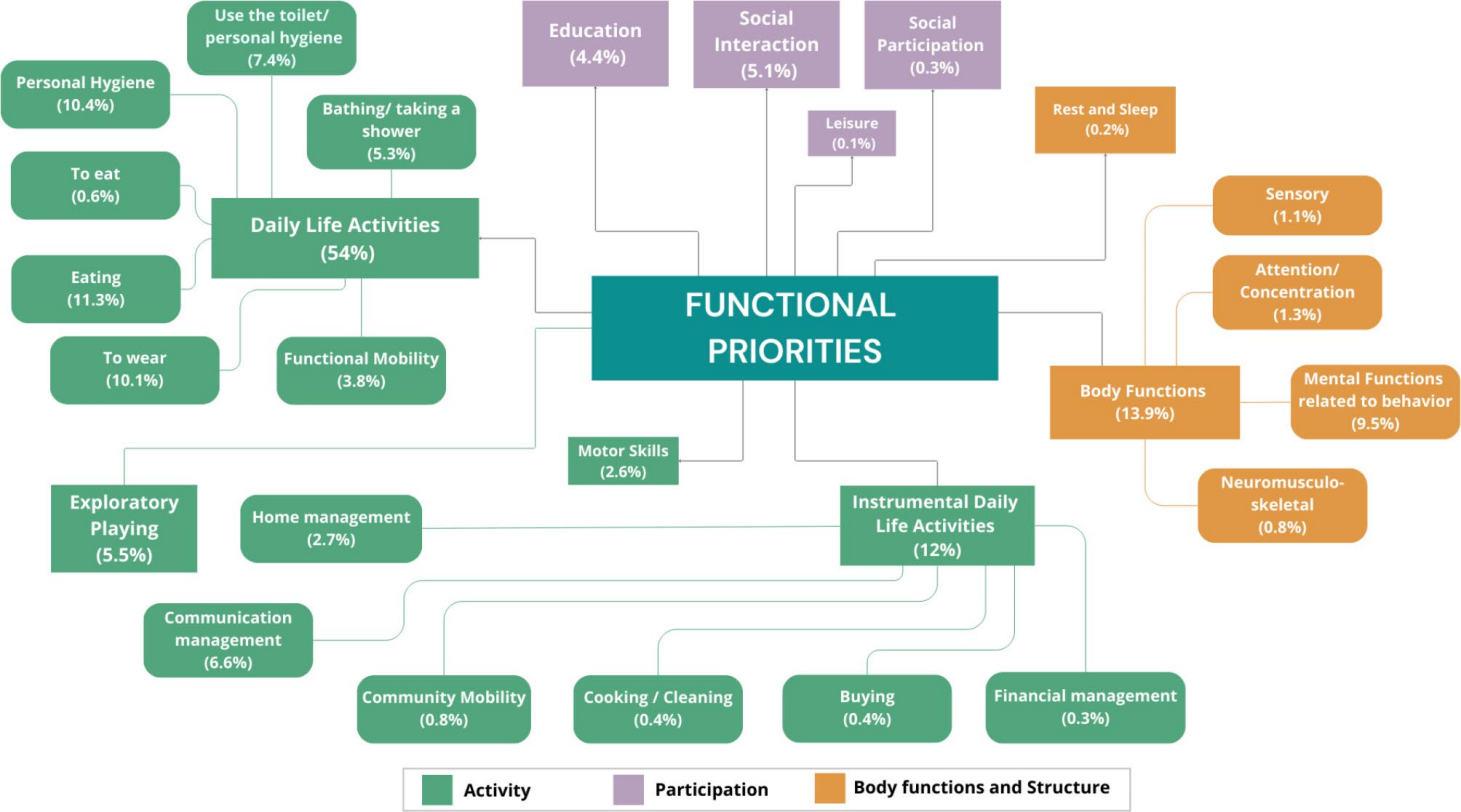
Functional priorities of parents of children and adolescents with ASD

A total of 1,203 functional priorities were established by 241 parents of children/adolescents with ASD using COPM. The most frequent priority was activities of daily living (54%). Among the priorities related to activities of daily living, the most frequent were eating (11.3%), personal hygiene (10.4%), and dressing (10.1%). For instrumental activities of daily living, the most frequent priority was associated with communication (6.6%). Regarding body function, parents listed mental functions related to behavior most frequently (9.5%). Approximately 5% of parents set goals related to social interactions. Priorities related to sleep, social participation, and leisure were the least frequent with percentages below 1%. Figure 2 presents an overview of the profile of functional priorities established by parents of children and adolescents with ASD. Figure 3.

Table 2 presents the functional priorities of parents of children and adolescents with ASD based on their level of support. A total of 278 functional priorities were established by parents of children/adolescents at support level 1, while parents of participants at levels 2 and 3 established 438 and 359 functional priorities, respectively. The priorities with the highest frequency of demand from parents at all levels of support included activities of daily living. Analysis with the chi-square test showed significant differences between different levels of support in only four of the 24 functional priorities established by parents (χ^2^ = 91.31; *p* = 0.01). Parents of children/adolescents classified at level 3 of support established priorities related to functional mobility and played more frequently than parents of children/adolescents classified at level 1 of support (*p* < 0.05). Meanwhile, demands related to education and management/establishment of the home were more frequent for parents of children/adolescents at level 1 than at level 3 (*p* < 0.05).

**Table 2:** Functional priorities of parents of children and adolescents with ASD by level of support

AOTA categories	AOTA subcategories	Support 1	Support 2	Support 3
		(*n* = 56)	(*n* = 88)	(*n* = 72)
		*n* (%)	*n* (%)	*n* (%)
Activities of daily living	Dressing	28 (10.1%)	51 (11.6%)	28 (7.8)
	Personal hygiene	32 (11.5%)	41 (9.4%)	39 (10.9)
	Feeding	26 (9.4)	44 (10%)	46 (12.8)
	Use the toilet and perform intimate hygiene	20 (7.2%)	35 (8.0%)	21 (5.8%)
	Bathing and bathing in the shower	13 (4.7%)	32 (7.3%)	14 (3.9%)
	Functional Mobility	5 (1.8%)	14 (3.2%)	21 (5.8%)
	Eating and swallowing	0	2 (0.5%)	3 (0.8%)
Instrumental activities of daily living	Communication management	16 (5.8%)	27 (6.2%)	31 (8.6%)
	Establishment and management of the home	16 (5.8%)	9 (2.1%)	6 (1.7)
	Financial management	3 (1.1%)	0	0
	Meal preparation and cleanup	1 (0.4%)	2 (0.5%)	2 (0.6%)
	Shopping	1 (0.4%)	1 (0.2%)	0
	Mobility in the community	1 (0.4%)	3 (0.7%)	4 (1.1%)
Functions of the body	Mental functions associated with behavior	30 (10.8%)	40 (9.1%)	42 (11.7%)
	Neuromusculoskeletal functions	3 (1.1%)	5 (1.8%)	2 (0.6%)
	Sensory functions	4 (1.4%)	4 (0.9%)	4 (1.1%)
	Attention and concentration functions	2 (0.7%)	9 (2.1%)	3 (0.8%)
Play	Exploratory or participatory play	9 (3.2%)	20 (4.6%)	29 (8.1%)
Social interaction	Social interaction with peers and family	16 (5.8%)	28 (6.4%)	12 (3.3%
Motor skills	Manual and fine motor skills	6 (2.2%)	13 (3.0%)	8 (2.2%)
Education	Formal educational participation	18 (6.5%)	23 (5.3%)	8 (2.2%)
Rest and sleep	Rest or Sleep preparation	0	1 (0.2%)	1 (0.3%)
Social Participation	Community and family participation	3 (1.1%)	1 (0.2%)	0
Leisure	Exploratory leisure	1 (0.4%)	0	0

Legend: AOTA, American Occupational Therapy Association

Table 3 shows the functional priorities of parents of children and adolescents with ASD according to age group. Parents of children aged 0 to 6 years established 544 functional priorities. Parents of children aged 7 to 12 years and adolescents (13 to 18 years) established 422 and 109 functional priorities, respectively. As observed in the results of the analyses by level of support, activities of daily living were mentioned most frequently by parents. Significant differences in the priority profile between age groups were found only for two of the 24 priorities established by parents (χ^2^ = 93.39; *p* < 0.01). Communication-related demands were significantly more frequent in children aged 0 to 6 years than in children aged 7 to 12 years or adolescents (*p* < 0.05). On the contrary, demands related to meal preparation and cleaning were significantly more frequent among adolescents’ parents than among children (*p* < 0.05).

**Table 3:** Functional priorities of parents of children and adolescents with ASD by age group

AOTA categories	AOTA subcategories	0 to 6 years (*n* = 102)	7 to 12 years (*n* = 85)	13 to 18 years (*n* = 19)
		*n* (%)	*n* (%)	*n* (%)
Activities of daily living	Feeding	73 (12)	48 (10.3)	14 (11.3)
	Dressing	59 (9.7)	54 (11.6)	8 (6.5)
	Personal Hygiene	61 (10)	47 (10.1)	17 (13.7)
	Functional Mobility	25 (4.1)	12 (2.6)	9 (7.3)
	Use the toilet and perform intimate hygiene	49 (8)	34 (7.3)	6 (4.8)
	Bathing and bathing in the shower	28 (4.6)	30 (6.4)	6 (4.8)
	Eating and swallowing	2 (0.3)	3 (0.6)	2 (1.6)
Instrumental activities of daily living	Communication management	53 (8.7)	23 (4.9)	3 (2.4)
	Establishment and management of the home	14 (2.3)	11 (2.4)	7 (5.6)
	Mobility in the community	3 (0.5)	6 (1.3)	1 (0.8)
	Meal preparation and cleanup	0	1 (0.2)	4 (3.2)
	Shopping	0	2 (0.4)	0
	Financial Management	0	1 (0.2)	2 (1.6)
Functions of the body	Mental functions associated with behavior	60 (9.8)	45 (9.6)	8 (6.5)
	Neuromusculoskeletal functions	3 (0.5)	5 (1.1)	1 (0.8)
	Sensory functions	9 (1.5)	3 (0.6)	1 (0.8)
	Attention and concentration function	5 (0.8)	6 (1.3)	4 (3.2)
Play	Exploratory or participatory play	42 (6.9)	23 (4.9)	1 (0.8)
Social Interaction	Social interaction with peers and family	27 (4.4)	31 (6.6)	3 (2.4)
Education	Formal educational participation	20 (3.3)	26 (5.6)	7 (5.6)
Motor Skills	Manual and fine motor skills	16 (2.6)	10 (2.1)	2 (1.6)
Rest and Sleep	Rest or Sleep preparation	1 (0.2)	2 (0.4)	0
Social Participation	Community and family participation	1 (0.2)	1 (0.2)	2 (1.6)
Leisure	Exploratory leisure	1 (0.2)	0	0

Legend: AOTA, American Occupational Therapy Association

When the functional priorities established by parents of children and adolescents with ASD were grouped into ICF domains, the results showed that 64.3% were associated with the ICF activity domain and 20.4% were associated with participation. Only 14.9% of functional priorities were associated with body structure and function domains.

### Effects of the MIG program on functional priorities

Table 4 details the percentage of functional priorities that showed changes in the COPM after the intervention program. According to parents’ perceptions, the percentage of functional priorities that improved by at least one point in COPM performance ranged from 13% (Mental functions related to behavior) to 80.9% (Communication management). In terms of satisfaction, this variation ranged from 26% (Dressing) to 78.74% (Communication management). Analyses with the paired samples Student’s t-test revealed significant effects in the pre- and post-intervention comparisons, both for performance (t[94] = 6.75; *p* < 0.01, d = 0.87) and for satisfaction (t[94] = 6.81; *p* < 0.01, d = 0.67).

**Table 4:** Percentage of functional priorities that achieved changes in COPM after the intervention

Functional priorities	Performance											Satisfaction									
	9	8	7	6	5	4	3	2	1	C%		9	8	7	6	5	4	3	2	1	C%
Feeding	0	0	2.60	3.47	4.34	14.78	12.17	8.69	18.26	63,64		1.73	1.73	2.60	6.95	6.08	6.08	12.17	13.91	9.56	60.88
Personal Hygiene	0	0.90	6.36	3.63	11.81	6.36	5.45	17.27	14.54	61,82		0	2.27	0	7.27	8.19	10.90	12.72	15.45	10.00	67.28
To wear	0	0	0	0	2.00	1.00	4.00	2.00	7.00	17.00		0	0	0	0	2.00	1.00	2.00	4.00	17.00	26.00
Mental functions related to Behavior	0	0	0	0	0	0	1.00	6.00	6.00	13.00		0	0	0	0	0	0	2.00	11.00	20.00	33.00
Use the toilet and perform personal hygiene	1.33	1.33	5.33	4.00	0	12.00	5.33	9.33	25.33	64,01		6.67	0	4.00	4.00	2.66	5.33	12.00	6.67	12.00	53.34
Communication Management	0	2.81	7.04	4.22	12.67	12.67	8.45	12.67	19.71	80,29		4.22	7.04	4.22	7.04	11.26	9.85	5.63	16.90	12.67	78.74
Exploratory playing	1.88	0	3.77	5.66	9.43	16.98	11.32	18.86	3.77	66,05		3.77	0	3.77	3.77	5.66	13.20	11.32	15.09	11.32	67.94
Bathing and taking a shower	0	0	1.85	1.85	1.85	11.11	1.85	1.85	12.96	56,71		0	1.85	0	1.85	7.40	5.55	11.11	18.51	9.25	55.57
Social Interaction with peers and family	3.92	5.88	1.96	3.92	3.92	17.64	13.72	7.84	11.76	70.60		0	3.92	1.96	7.84	9.80	9.80	11.76	11.76	17.64	74.52
Formal education participation	0	2.17	2.17	6.52	0	0	13.04	15.21	10.86	60.80		4.34	0	2.17	4.34	2.17	15.21	13.04	4.34	19.56	65,32

Table 4 shows change in Canadian Occupational Performance Measure scores percentage (performance and satisfaction) according to the activity-based goals achieving intervention effect. To interpret the data, verify the functional priority rows and then the percentage of children and adolescents who changed scores according to performance and satisfaction scores column. Note: COPM: Canadian Occupational Performance Measure; C% = Cumulative percentage of functional priorities that achieved an improvement of 1 point or more in COPM

## Discussion

This study aimed to describe the functional priorities of families of Brazilian children and adolescents with ASD, secondarily, to evaluate changes in the performance and satisfaction of these functional priorities after intervention with the MIG program. To the best of our knowledge, this is the first study to investigate functional priorities among Brazilian children and adolescents with ASD. Our findings indicate that activities of daily living, followed by behavioral difficulties, communication, play, and social interaction, are the main functional priorities in parents’ perceptions. In general, functional priorities were similar among different levels of support and age groups. The results also showed that most functional priorities were classified in the activity domain of the ICF (64%). Finally, the findings of this study showed that the MIG program was perceived as effective for most of the functional priorities of children/adolescents with ASD, with perceptions of significant improvements in performance and satisfaction after three months of intervention.

The findings of this study revealed that activities of daily living, especially eating, dressing, and personal hygiene, are the main functional priorities of parents of children and adolescents with ASD. This finding is intriguing because it was expected that the priorities of parents would be directly associated with the triad of central symptoms of ASD, that is, difficulties in social interaction, communication, and restricted interests. However, this finding is consistent with psychological theories that suggest that individuals first seek to satisfy their basic daily needs and then consider personal achievements and leisure activities [25]. These theories also explain the low number of priorities for leisure and social participation found in this study. Our results suggest that parents of children/adolescents with ASD want, above all, their children to gain independence in self-care. There is evidence that children and adolescents who can meet their basic self-care needs may have better socialization skills with peers, greater participation in community activities, and greater independence in adult life [19].

Studies in developed countries investigating the functional priorities of parents of children and adolescents with ASD have shown that social and communication skills are the main priorities. For example, a UK study involving 350 parents of children with ASD identified social relationships as a high priority for parents [26]. Similarly, a study involving 45 parents in North American children with ASD identified social and communication skills as the highest treatment priority [27]. Our results involving Brazilian children with ASD differ from these results by highlighting activities of daily living as the main priority of parents. Our results are similar to those of a recent study that involved a sample from an underdeveloped country. Beheshti et al. [28] showed that the first priority for mothers of Iranian children with ASD was self-care. These findings point to a possible difference in the functional priorities of parents of children with ASD in developed and underdeveloped countries. A possible explanation for these data is that, in underdeveloped countries, the lack of financial resources and other public health issues, as well as the difficulty in accessing appropriate and early interventions, can hinder the rehabilitation and clinical evolution of children and adolescents with ASD. In support of this hypothesis, 70% of the children in these studies were classified into levels 2 and 3 of support, indicating the need for greater support in activities of daily living. Improvements in the performance of children and adolescents in basic self-care activities reflect greater independence, autonomy, and efficiency in carrying out these activities, which reduces parental overload and stress. This partially explains the greater interest of parents in activities of daily living.

The results of this study showed that social participation was not a priority for parents of children and adolescents with ASD, which only made 0.2% of the goals. The hypothesis was raised that children can already participate satisfactorily, which justifies the low number of priorities related to social participation. However, this hypothesis is not supported by the literature. Children with ASD have restricted social participation [29]. These children participate in activities less frequently and with fewer variations than their typical peers [30]. A recent study involving Brazilian children with various disabilities showed that participation occurs on average only twice in 4 months [31]. It is possible that the parents in the present study were only concerned with the basics (i.e., activities of daily living) and did not see participation as something possible for their children. This finding is concerning because social participation is one of the most important predictors of physical and mental health in children [32]. According to the World Health Organization, social participation promotes self-esteem and facilitates the development, health and well-being of children [33]. Consistent with this, community participation has been shown to improve life satisfaction and well-being and reduce loneliness in children with ASD [34]. There is strong evidence that interventions that involve social skill groups, joint attention, and parental-mediated strategies can improve social participation in people with ASD [35]. Despite this evidence, more participation-focused intervention programs are required for children and adolescents with ASD.

In general, the functional priority profiles found in the present study were similar between the different levels of support for ASD. Some exceptions were priorities related to functional mobility and play, which were more frequent in level 3 support. This finding is not surprising because children and adolescents with ASD at level 3 of support have more severe impairments, which may indicate co-occurring conditions that affect their mobility, beyond the core features of autism [10]. Regarding playing, the greater demand reported by parents of children/adolescents at level 3 of support is in line with studies involving children with cerebral palsy. In a study by Brandão, Oliveira and Mancini [36], demands related to playing were more frequent in children with greater motor impairments (GMFCS level V). This finding highlights the importance of introducing play into therapeutic intervention, given its importance in the global development of children [37]. As observed in the analyses of the priority profiles between support levels, a similar pattern was observed for age groups. An exception was communication-related priorities, which were more frequent in children aged < 6 years. This finding was also not surprising since this is a skill acquired in the first years of life, making parents create expectations for the achievement of communication skills.

When the functional priorities of parents of children and adolescents with ASD were grouped into ICF categories, the results showed that most of the priorities were within the activity category, followed by participation. A small number of priorities were associated with body structure and function. This result is explained by the large number of recent studies that have emphasized the importance of interventions focusing on activity and participation, thus minimizing the relevance of interventions focused on body structure and function [38]. Promoting activity and participation is important. However, it does not diminish the importance of managing and preventing damage to bodily structures and functions [39]. According to Rosenbaum and Stewart [5], we should not diminish the importance of interventions based on deficiencies in bodily structures and functions because interventions in all elements of the ICF model can be important, appropriate, and interconnected.

Most deficits in body structure and function in ASD patients are motor deficits. The American SPARK study, involving 11,814 children with ASD, revealed that about 80% of these children have motor difficulties, yet only 32% receive physical therapy [40]. This discrepancy is due to misinformation about the benefits of physical therapy, geographical and logistical access difficulties, high costs, and insufficient health insurance coverage. Additionally, families and professionals tend to prioritize other interventions, such as behavioral and communication therapies [41]. The lack of specialized physical therapists and social stigma reinforce the perception that physical therapy is not a priority. Behavioral challenges and sensory issues can hinder adherence to physical therapy sessions, exacerbating access to these essential services. Motor problems in ASD represent a significant barrier to daily activities and social and cognitive development, being related to difficulties in the social domain [42–44]. It is unknown whether motor deficits are a co-occurrence or comorbidity of ASD, and changes in the clinical process are necessary to address these issues from the recognition phase to diagnosis and intervention.

The study results indicate that, although parents of children and adolescents with ASD prioritize ADLs in treatment, most traditional intervention programs primarily focus on developing behavioral and social communication skills. Interventions such as Naturalistic Developmental Behavioral Interventions (NDBI) and the Early Start Denver Model (ESDM) tend to focus more strictly on behavioral or communication changes, revealing a discrepancy between parental priorities and conventional therapeutic emphasis [45, 46]. This finding underscores the importance of ADLs in the context of pediatric treatment and highlights the need to develop comprehensive and interprofessional interventions that address all the needs of children and adolescents with ASD and their families in a balanced manner. To align interventions with family needs, professionals must integrate family-centered approaches into their clinical practice, incorporating strategies that meet the needs and priorities of parents [10, 11], as exemplified by the MIG program [8]. Interventions that holistically address family needs not only enhance therapeutic efficacy but also strengthen the collaboration between professionals and caregivers, an essential element for success in child development.

The management of ASD presents substantial challenges for both families and healthcare professionals, largely due to the geographical dispersion of necessary treatments, which are often provided at multiple locations. This fragmentation increases the complexity of the therapeutic process and intensifies the burden on families, who must balance constant travel with integrating therapy sessions into their daily routines. In this context, the MIG program offers an innovative solution by consolidating all interventions into a single centralized location [8]. Although this centralization may not address every specific need of children with ASD, it provides considerable advantages, such as reducing transportation costs and saving time, thus alleviating the logistical pressures faced by families. Furthermore, this integrated approach enhances access to services and facilitates more efficient care coordination, potentially improving therapeutic outcomes and the quality of life for the families involved.

This study reveals significant progress in functional goals and parental satisfaction, indicating effective collaboration between family and professionals. The theoretical model guiding the MIG program emphasizes family-centered practice, highlighting the crucial role of active family involvement in the rehabilitation process [9–11, 13]. This includes joint goal setting and personalized adaptation of interventions. The situated learning theories supporting this model suggest that skill transfer is more effective when interventions occur in environments that mirror natural contexts, such as the “City of Tomorrow” [8]. Aligned with the ICF, the MIG program not only facilitates the practical application of skills learned in clinical settings to everyday life but also adopts a comprehensive approach that considers the specific needs and preferences of each family. The integration of these theoretical principles may explain the high level of satisfaction observed among parents, reinforcing the effectiveness of family-centered practices in pediatric rehabilitation. To deepen this analysis, future research could explore how these theoretical principles affect parental perception and the outcomes achieved with therapeutic interventions.

The findings of this study should be interpreted with caution due to some limitations. The sample was selected by convenience and the participation of children and adolescents with ASD and their parents was voluntary. It is possible that the priorities of the parents who did not choose to participate in this study were different. Furthermore, the parents participating in the present study were assisted by health plans and the findings may not be generalizable to the population using the Unified Health System (Sistema Único de Saúde - SUS). Finally, an important limitation of this study is the geographical distribution of participants, primarily concentrated in the Southeast (61%) and Midwest (29%) regions, with significantly lower representation from other regions of Brazil, such as the North (4.6%), South (4.1%), and Northeast (1.2%). This geographical concentration may have influenced the results, as the functional priorities of families can vary according to regional factors, such as cultural, socioeconomic differences, and access to healthcare services. Therefore, this uneven distribution may limit the generalization of the findings to the entire Brazilian population. It is important to consider that the reported perceptions and priorities may more specifically reflect the experiences and contexts of the regions most represented in the study.

Although valid and reliable [20], the COPM assesses changes in the child/adolescent’s performance based on the parents’ subjective perception. Future studies should include instruments that objectively evaluate the impacts of the MIG program in the different domains of the ICF. No control group was included in this study, which prevented a direct link between the intervention and documented functional improvements in the study participants. In addition to the control group, researchers may also consider long-term follow-up. No follow-up measures were taken, making it impossible to assert whether the gains achieved would be sustained after the conclusion of the intensive intervention.

## Conclusion

Interventions aimed at improving activities of daily living, behavioral difficulties, communication, play, and social interaction appear to be the highest priority for parents of Brazilian children and adolescents with ASD. The alignment between parental functional priorities and therapeutic efforts is critical to reducing parental stress and improving treatment outcomes. The findings of this study are useful and should be used by professionals and policy makers to implement intervention strategies capable of meeting these needs. The MIG program demonstrated to be able to improve performance and satisfaction for most of the functional priorities of parents of children/adolescents with ASD in this study. However, future studies that include objective measurements and a control group are still needed.

## Data Availability

The data supporting this research are available by contacting the authors.

## Abbreviations

ASD: Autism Spectrum Disorder
COPM: Canadense Occupational Performance Measure
MIG: Global Integration Method
MIG Flex: Flexible Therapeutic Suit
AOTA: American Occupational Therapy Association
ICF: International Classification of Functioning, Disability, and Health
